# Fatal Vertebral Artery Injury in Penetrating Cervical Spine Trauma

**DOI:** 10.1155/2015/571656

**Published:** 2015-11-12

**Authors:** Chadi Tannoury, Anthony Degiacomo

**Affiliations:** ^1^Boston University Medical Center, 840 Harrison Avenue, Dowling 2 North, Orthopaedic Administration, Boston, MA 02118, USA; ^2^Boston Medical Center, Boston, MA 02118, USA

## Abstract

*Study Design*. This case illustrates complications to a vertebral artery injury (VAI) resulting from penetrating cervical spine trauma. *Objectives*. To discuss the management of both VAI and cervical spine trauma after penetrating gunshot wound to the neck. *Summary of Background Data.* Vertebral artery injury following cervical spine trauma is infrequent, and a unilateral VAI often occurs without neurologic sequela. Nevertheless, devastating complications of stroke and death do occur. *Methods*. A gunshot wound to the neck resulted in a C6 vertebral body fracture and C5–C7 transverse foramina fractures. Neck CT angiogram identified a left vertebral artery occlusion. A cerebral angiography confirmed occlusion of the left extracranial vertebral artery and patency of the remaining cerebrovascular system. Following anterior cervical corpectomy and stabilization, brainstem infarction occurred and resulted in death. *Results*. A fatal outcome resulted from vertebral artery thrombus propagation with occlusion of the basilar artery triggering basilar ischemia and subsequent brainstem and cerebellar infarction. *Conclusions*. Vertebral artery injury secondary to cervical spine trauma can lead to potentially devastating neurologic sequela. Early surgical stabilization, along with anticoagulation therapy, contributes towards managing the combination of injuries. Unfortunately, despite efforts, a poor outcome is sometimes inevitable when cervical spine trauma is coupled with a VAI.

## 1. Introduction

Vertebral artery injury (VAI), though initially believed to be unusual, has been found in higher frequency following cervical spine trauma. In the current literature, injury to the vertebral arteries was noticed to be associated with blunt cervical trauma, namely, fracture extending through the transverse foramen or facet dislocation with/without fracture. The reported incidence of VAI subsequent to cervical spine trauma is variable and ranges from 0.53 to 88% [1, 2]. In a study by Mueller et al. [1], the incidence of VAI was 27.5% in cervical spine injuries with transverse foramen fracture and/or facet dislocation. In a large study, Sanelli et al. [3] identified an incidence of 0.53% of VAI in all blunt trauma admissions with cervical spine injuries present in 71% of these injuries.

The most common mechanism of VAI is a motor vehicle accident where a hyperextension moment, accompanied with/without lateral flexion or rotation, results in a closed injury to the vertebral arteries. Less commonly, a fall or pedestrian motor vehicle accident with a cervical spine injury results in VAI. The vertebral artery, a branch of the subclavian arteries, ascends through the neck via the transverse foramen of the cervical spine before combining with each other to form the basilar artery. Along this ascension in the neck, the vertebral artery is most vulnerable to injury at the entry point into the C6 transverse foramen and at the exit point at the atlas-axis junction. Within the transverse foramen, the vertebral artery can occupy as little as 8% or as much as 85% of the foramen [3]. In the majority of individuals, 50% have a dominant left vertebral artery, 25% dominant right vertebral artery, and 25% nondominant vertebral arteries [2]. VAI is most commonly unilateral. An individual can sustain a unilateral injury to a vertebral artery without suffering from neurologic sequela.

Devastating complications of stroke and death can be a consequence of VAI. Sanelli et al. found a stroke rate of 24% and an 8% death rate attributable to VAI [3]. Formative factors, determining risk of stroke, are the patency and flow within the contralateral vertebral artery, circle of Willis, and carotid arteries. Initially, patients with VAI can be asymptomatic and have no neurologic deficits on presentation. In a study by Biffl et al., a time interval of 18 hours elapsed between time of injury and neurologic symptoms in 44% of cases [4]. Stroke, resulting from VAI, affects the posterior circulation with subsequent clinical manifestations of vertebrobasilar ischemia. In a study by Mueller et al., 15.7% had PICA infarctions following VAI [1]. In additional studies, most of the subjects suffering from strokes were those who sustained left-sided VAI [1, 2]. Cervical spine injury, coupled with VAI, is a dangerous combination with a reported mortality rate of 40% [5].

**Figure 1:**
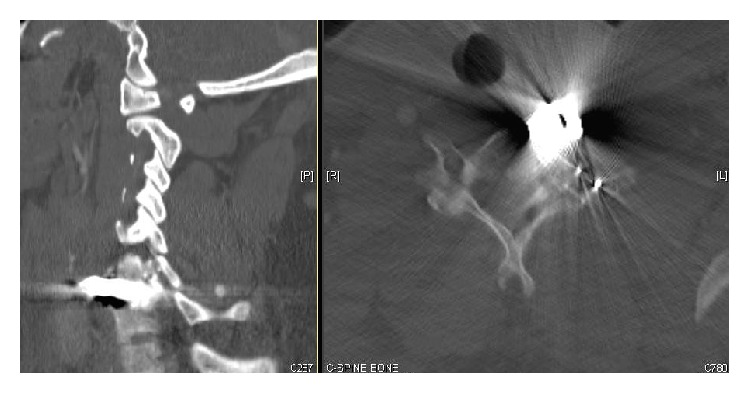
Cervical spine CT scan (sagittal and axial cuts) showing the bullet along with the C6 vertebral body and lateral mass fractures.

**Figure 2:**
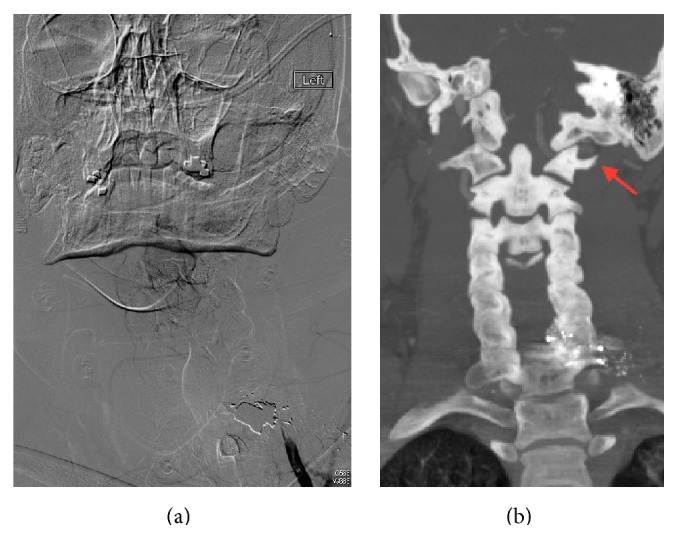
(a) Angiography showing occlusion of the left vertebral artery. (b) CT angiogram showing reconstitution of the left vertebral artery (red arrow).

This report will present the case of a 21-year-old male who sustained a gunshot wound with entry over the left scapula and terminating in the C6 vertebral body. This passage of the missile resulted in a C6 vertebral body fracture, C6 left lateral mass fracture, C5 and C7 left transverse foraminal fractures, and C6-C7 left facet fracture subluxation (Figure 1). Along with these fractures, from the blast injury of the missile, the left vertebral artery was thrombosed (Figure 2(a)) with subsequent migration of the thrombus to the basilar artery resulting in brainstem and cerebellar infarcts and ultimately death.

## 2. Case Report

A 21-year-old male sustained a single gunshot wound with entry over the left scapula. At the scene, the emergency medical response personnel found him on the ground alert, unable to move his legs, and noting pain all over his body. He was hemodynamically stable in the field. Ground ambulance immobilized the neck with a cervical collar and brought him to a local level 1 trauma center. At arrival to the trauma bay, he had a Glasgow coma scale score of 15. He was primarily complaining of bilateral hand numbness. On further examination, he had no motor or sensory function present in the lower extremities. In the upper extremities, sensation was present but diminished from T1 and above, with no sensation below T1. Motor function in the upper extremities was graded 3 (based on ASIA impairment scale) in all the key muscles on the right and only grade 2 on the left in the deltoid and biceps muscles with no motor further distal. Next, he was taken to the computed tomography (CT) suite for imaging of the head, neck, and thorax. Imaging, from the CT scan, revealed bullet tract through the posterior lateral left upper hemithorax, left scapular body, left lung apex, and base of left neck with bullet fragments terminating in the C6 vertebral body. Initial studies showed no acute intracranial abnormalities; however, there were fractures of the left C5 and C7 transverse foramina and processes and left C6 vertebral body and lamina fractures with bony and bullet fragments in the left aspect of the spinal canal at the C6 level (Figure 1). As a result, there was a dense left epidural hematoma within the spinal canal at C2 through C7 levels. At the time, CT neck angiogram showed that the left extracranial vertebral artery was occluded, beyond 1 cm from its origin. Subsequently, the patient underwent angiography to further assess the cerebral vascular supply. On the angiogram, occlusion of the proximal portion of the left vertebral artery was noted with reconstitution in the distal portion of the artery near the occipitocervical junction (Figure 2(b)). No contrast extravasation from the left vertebral artery was noted. Patency was noted in the right vertebral artery and bilateral common, internal, and external carotid arteries. Vascular surgery specialists, after conducting and reviewing the angiogram, determined that no further intervention was required as the right vertebral artery along with bilateral internal carotid arteries was patent.

Following the imaging studies and angiogram, the patient was admitted to the surgical intensive care unit (SICU) for continuous neurologic and hemodynamic control and observation. On the next hospital day, the patient was optimized by the vascular surgery team and the surgical intensive care unit trauma team and was deemed stable to proceed forward with surgical intervention of the spine. Given the spinal cord injury and preparation for spinal surgery, anticoagulation was not started prior to reporting to the operating room. The goals of the surgery were to decompress the cervical spinal cord and to provide stability to the spinal column.

**Figure 3:**
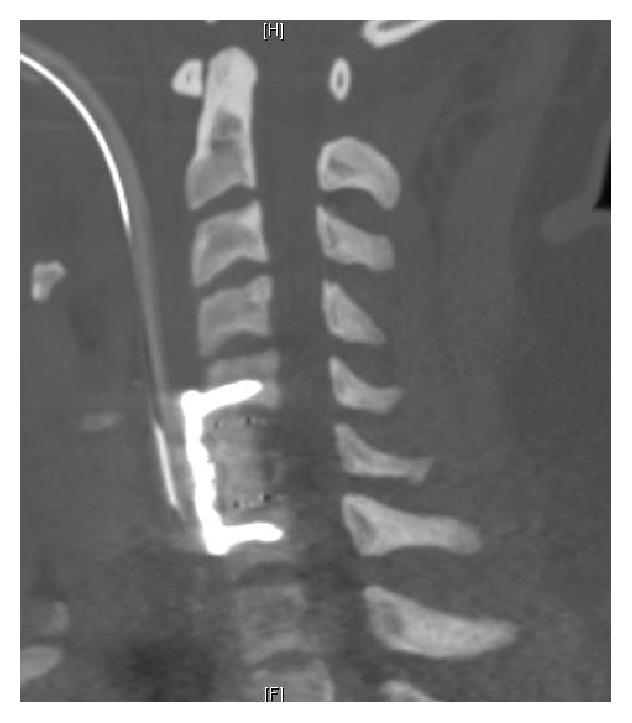
Postsurgical CT scan showing the corpectomy cage and the anterior cervical plate and screws fixation.

The patient received a successful awake fiber-optic intubation with protection of the cervical spine and then general anesthesia was induced. Via a standard left sided Smith-Robinson approach, anterior cervical corpectomy of C6 and anterior cervical decompression were performed within C5–C7. Bony and bullet fragments were removed from within the canal and following decompression, and while avoiding excessive distraction, the anterior column was reconstructed using an appropriately sized interbody cage device packed with autologous bone graft. Anterior cervical plate and screws were used to provide stability to the cage-graft construct and maintain the proper cervical alignment (Figure 3). Following the procedure, the patient remained intubated for airway edema concerns. The sedation was weaned after transporting the patient to the SICU from the operative suite; however, he remained unarousable. Given the neurologic change in the patient status, an emergent head CT scan was conducted and showed an acute basilar infarct.

**Figure 4:**
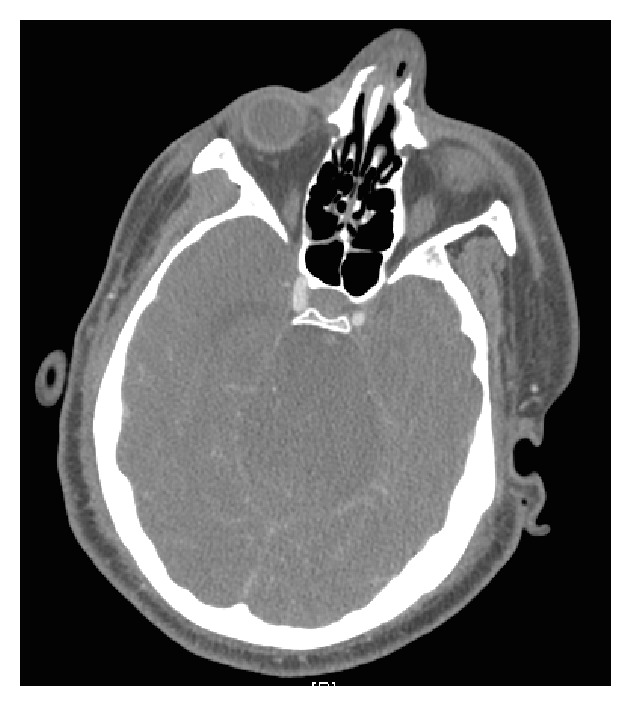
Head CT scan showing brainstem edema after infarct.

Head and neck CT angiogram were subsequently performed and showed new basilar artery segmental occlusion consistent with migration of a thrombus from the distal left vertebral artery. Both neurology and neurosurgery teams recommended hyperosmolar treatment, as the patient was not considered a good candidate for revascularization. Twelve hours later, repeat head CT scan showed progressive hydrocephalus and brainstem and cerebellar infarcts with impending herniation (Figure 4). The patient was subsequently diagnosed with brain death and, with consent from his family, care was withdrawn.

## 3. Discussion

Cervical spine trauma, which includes a VAI, is a combined diagnosis that carries profound morbidity and mortality. Although the combined injuries are recognized more frequently, the clinical presentation may still be obscure and missed if there is no heightened index of suspicion. Clearly, on presentation of this case, the patient had signs and symptoms of spinal cord injury, but initially no clinical signs pertaining to vertebrobasilar ischemia. Clinical signs of vertebrobasilar ischemia, present as altered consciousness, dysphagia, dysarthria, diplopia, vertigo, and nystagmus, among others. Given the mechanism behind this traumatic case, proper additional imaging was acutely performed. Following the CT scans of the head, neck, and thorax, which showed left C5 and C7 transverse process/foraminal fractures, an angiogram of the neck was performed revealing thrombosis, rather than transection, of the left vertebral artery. Importantly, by means of collateral circulation via the contralateral vertebral artery, unilateral occlusion of the vertebral artery rarely results in neurologic sequela. In a study by Friedman et al., 20% of unilateral occlusion of the vertebral artery manifested clinically upon presentation [6]. On the other hand, bilateral vertebral artery injuries or disruption further downstream the posterior basilar cerebral circulation becomes discernibly evident upon insult. Wirbel et al., in a case report, describe the fatal outcome following bilateral vertebral artery injury after dislocating cervical spine trauma [7]. Yet when unilateral VAI result in neurologic deficits the physical manifestation is not initially apparent. Golueke et al. reported that 26% of patients, with vertebral artery injury, developed major neurologic deficits directly attributable to missile injury; however, most of these deficits became evident in a delayed fashion [8]. In a study by Biffl et al., the mean time from injury to stroke was 4.3 days with 78% of strokes occurring more than 48 hours after injury [2]. Multiple explanations behind this delay have been theorized including thrombus progression, cerebral swelling, or incremental evolution of the vascular injury with swelling and development of flap, dissections, or pseudoaneurysm. Evidently, arterial injury resulting in flap, dissection, or even pseudoaneurysm, rather than complete occlusion by thrombus, has a greater risk of embolic stroke. Even more disturbing than the stroke ramification from VAI is the direct mortality attributed to the vascular vertebral injury. In our case, the final outcome was death from the succession of VAI to basilar occlusion to brainstem and cerebellar infarction. Biffl et al. reported an 8% death rate directly attributable to VAI [2].

Cervical spine injury is highly dependent on the mechanism of injury. In a study by Rhee et al., the highest rates of cervical spine fracture and cervical spinal cord injury were found in patients sustaining gunshot wounds, as opposed to blunt assault or stab wounds [9]. Furthermore, these cervical spine injuries, as a result of low velocity missiles, uncommonly have progressive neurological deficits and these deficits are unaffected by surgery [10]. In consideration of all blunt traumas, VAI has a reported incidence rate ranging from 0.24% to 2% [11–13]. The majority of VAI, sustained from blunt trauma, is associated with cervical spine trauma [2]. In fact, Biffl et al. reported that the only independent risk factor for blunt VAI was cervical spine injury [2]. Specifically, cervical spine injuries with subluxation/dislocation, fracture involving transverse foramen, or fracture involving the upper cervical spine are highly associated with VAI [1, 2]. Mueller et al. reported a 20% incidence of VAI in fractures involving the transverse foramen and a 31% incidence of VAI in cervical subluxations/dislocations [1]. Additional studies report that the most common cause of closed traumatic vertebral artery occlusion is a flexion distraction injury to the cervical spine [14]. In our case, the patient sustained a penetrating gunshot wound to the neck, which did not directly transect the vertebral artery but rather resulted in vertebral body fracture accompanied by fracture of the transverse foramen and subsequent thrombosis of the vertebral artery.

At the entry and exit point of the transverse foramen, the vertebral artery is most susceptible to injury. The left vertebral artery is the dominant artery in 50% of individuals and injury to this dominant vessel carries a higher risk of neurologic insult [15]. Biffl et al. reported that 88% of posterior ischemic strokes occurred with left sided VAI [2]. In our case, the patient sustained an injury to the left vertebral artery with consequent progression of the thrombus to the basilar artery. Diagnosis of arterial injuries caused by penetrating neck trauma can be adequately identified by helical CT angiography. In a study by Múnera et al., the accuracy of helical CT angiography, in detecting arterial injury sustained from penetrating trauma, demonstrated a sensitivity of 90%, specificity of 100%, positive predictive value of 100%, and negative predictive value of 98% [16]. Our patient's diagnosis of left VAI was identified on CT angiogram of the neck and successive neck angiography confirmed this occlusion of the left extracranial vertebral artery, along with additional information of reconstitution and patency of the remaining cerebral vasculature. As previously reported by Biffl et al., classification of cerebrovascular injuries is by grade: grade 1 is vessel injury with <25% luminal stenosis, grade II is vessel injury with ≥25% luminal stenosis, grade III is pseudoaneurysm, grade IV is vessel occlusion, and grade V is transection [17]. However, in a subsequent study by Biffl et al., grade of VAI was not associated with neurologic outcome [2]. The grade of VAI, sustained by our patient, was IV given the vessel occlusion by thrombus and this thrombus eventually propagated to the basilar vasculature.

Surgical fixation of unstable cervical spine injuries is performed to prevent further insult to the spinal cord and VAI. Heiden et al. reported no further development of neurologic deficits, sustained from penetrating gunshot to cervical spine, after surgical fixation [10]. In a subsequent study by Mueller et al., all patients with VAI and cervical spine injuries underwent external fixation and the follow-up of these patients showed no secondary dislocation and unremarkable neurologic status [1]. The cervical spine injury in our patient was deemed unstable; on hospital day 2, the patient underwent C6 corpectomy along with C5–C7 anterior decompression and fusion with internal fixation using anterior cervical plate and screws. Despite the aforementioned literature supporting internal and external fixation to prevent further neurologic deterioration, one may not exclude the surgical manipulation, of an unstable cervical spinal segment with vascular injury, as a trigger to subsequent neurovascular insult.

To prevent development of neurologic deficits, and injury propagation, antithrombotic therapy is recommended for management of VAI [13, 18]. In a study by Biffl et al., there was a trend towards decreased deterioration in neurologic status in VAI treated with heparin; 60% of patients not treated with heparin developed neurologic deterioration compared to 19% of patients treated with heparin [2]. Our patient, given his spinal cord injury and preparation for surgery, was not started on anticoagulation prior to reporting to the operating room. Despite anticoagulation, Mueller et al. reported that in patients with VAI 5.5% became clinically symptomatic and 2.9% died secondary to cerebrovascular ischemia [1].

## 4. Conclusion

The patient expired from brainstem infarction secondary to occlusion of the basilar artery following penetrating injury to the cervical spine. VAI secondary to cervical spine trauma can lead to potentially devastating neurologic sequela. Early surgical stabilization, along with anticoagulation therapy, can contribute towards managing the combination of injuries. Unfortunately, despite appropriate management, a poor outcome is sometimes inevitable when cervical spine trauma is coupled with a vertebral artery injury.
