# Corrigendum to “Fatal Vertebral Artery Injury in Penetrating Cervical Spine Trauma”

**DOI:** 10.1155/2017/3861804

**Published:** 2017-03-12

**Authors:** Chadi Tannoury, Anthony Degiacomo

**Affiliations:** ^1^Boston University Medical Center, 840 Harrison Avenue, Dowling 2 North, Orthopaedic Administration, Boston, MA 02118, USA; ^2^Boston Medical Center, Boston, MA 02118, USA

In the article titled “Fatal Vertebral Artery Injury in Penetrating Cervical Spine Trauma,” [1] a Disclosure should be added, and the Conflict of Interests should be corrected as follows.
